# Adjusting ferritin concentrations for inflammation: Biomarkers Reflecting Inflammation and Nutritional Determinants of Anemia (BRINDA) project

**DOI:** 10.3945/ajcn.116.141762

**Published:** 2017-06-14

**Authors:** Sorrel ML Namaste, Fabian Rohner, Jin Huang, Nivedita L Bhushan, Rafael Flores-Ayala, Roland Kupka, Zuguo Mei, Rahul Rawat, Anne M Williams, Daniel J Raiten, Christine A Northrop-Clewes, Parminder S Suchdev

**Affiliations:** 1 Strengthening Partnerships, Results, and Innovations in Nutrition Globally, Arlington, VA;; 2 Helen Keller International, Washington, DC;; 3 GroundWork, Fläsch, Switzerland;; 4 Department of Oncology, Johns Hopkins University, Baltimore, MD;; 5 University of North Carolina at Chapel Hill, Gillings School of Global Public Health, Chapel Hill, NC;; 6 Nutrition Branch, CDC, Atlanta, GA;; 7 Nutrition Section, UNICEF, New York, NY;; 8 Poverty Health and Nutrition Division, International Food Policy Research Institute, Dakar, Senegal;; 9 Emory University, Department of Pediatrics, Atlanta, GA;; 10 Pediatric Growth and Nutrition Branch, *Eunice Kennedy Shriver* National Institute of Child Health and Human Development, NIH, Bethesda, MD; and; 11 Independent Public Health Nutrition Consultant, Cambridge, United Kingdom

**Keywords:** anemia, ferritin, inflammation, iron deficiency, meta-analysis, nutritional assessment

## Abstract

**Background:** The accurate estimation of iron deficiency is important in planning and implementing interventions. Ferritin is recommended as the primary measure of iron status, but interpretability is challenging in settings with infection and inflammation.

**Objective:** We assessed the relation between ferritin concentrations and inflammation and malaria in preschool children (PSC) (age range: 6–59 mo) and women of reproductive age (WRA) (age range: 15–49 y) and investigated adjustment algorithms to account for these effects.

**Design:** Cross-sectional data from 15 surveys for PSC (*n* = 27,865) and 8 surveys for WRA (24,844), from the Biomarkers Reflecting the Inflammation and Nutritional Determinants of Anemia (BRINDA) project were analyzed individually and combined with the use of a meta-analysis. Several approaches were explored to estimate depleted iron stores (ferritin concentration <12 μg/L in PSC and <15 μg/L in WRA) in inflammation and malaria settings as follows: *1*) increase ferritin-concentration cutoff to <30 μg/L; *2*) exclude individuals with C-reactive protein (CRP) concentrations >5 mg/L or α-1-acid glycoprotein (AGP) concentrations >1 g/L; *3*) apply arithmetic correction factors; and *4*) use a regression correction approach.

**Results:** Depleted iron-store estimates incrementally increased as CRP and AGP deciles decreased (4% compared with 30%, and 6% compared with 29% from highest compared with lowest CRP deciles for pooled PSC and WRA, respectively, with similar results for AGP). Depending on the approach used to adjust for inflammation (CRP plus AGP), the estimated prevalence of depleted iron stores increased by 7–25 and 2–8 absolute median percentage points for PSC and WRA, respectively, compared with unadjusted values. Adjustment for malaria in addition to CRP and AGP did not substantially change the estimated prevalence of depleted iron stores.

**Conclusions:** Our results lend support for the use of internal regression correction to estimate the prevalence of depleted iron stores in regions with inflammation. This approach appears to mathematically reflect the linear relation of ferritin concentrations with acute-phase proteins. More research is warranted to validate the proposed approaches, but this study contributes to the evidence base to guide decisions about how and when to adjust ferritin for inflammation.

## INTRODUCTION

Iron deficiency remains an important public-health problem primarily in women and children (1–4). Iron deficiency has been estimated to affect 1.62 billion people worldwide, although these figures are approximations because of limited available estimates of iron deficiency globally (5). The accurate measurement of iron deficiency is important in the planning and implementation of interventions and for comparisons between countries or geographic areas.

The WHO currently recommends low ferritin concentrations (termed depleted iron stores) as the primary measure of population-level iron deficiency because it reflects body iron stores, has a long history of use with well-established laboratory methods and cutoffs, is easier to measure than more-invasive methods (e.g., bone marrow iron), and is responsive to interventions (6). However, in populations with high levels of inflammation, ferritin concentrations not only reflect nutritional iron but are also impacted by the acute-phase response (APR) (7). The APR is an immunologic process that causes certain acute-phase proteins (APPs) in the body to rise or fall in response to microbial invasion, tissue injury, immunologic reactions, and inflammatory processes (8). In addition to its role as a marker of iron stores, ferritin is a positive APP that becomes elevated during an APR. In an inflammatory state, ferritin synthesis appears to be upregulated by cytokines independent of iron homeostasis (9, 10). Ferritin concentrations may also increase in the presence of malaria infection potentially because of impaired iron incorporation or malaria-associated inflammation (11–13). Thus, not accounting for changes in the control mechanisms of iron during states of inflammation or malaria infection may translate to a significant underestimate of depleted iron stores (7, 14).

Interest has emerged in methods to account for inflammation and malaria infection in the interpretation of iron biomarkers (15–18). Because it is not possible to determine the level of inflammation in apparently healthy people with the use of clinical signs or symptoms, the WHO has recommended measuring other APPs, namely C-reactive protein (CRP) and α-1-acid glycoprotein (AGP), concurrently with ferritin to confirm the presence of an APR (6). The WHO has suggested measuring both of these biomarkers (6) because they reflect different stages of the APR (15).

Several approaches have been proposed about how to use CRP and AGP to account for inflammation and infections such as malaria to increase the sensitivity of detecting depleted iron stores at the population level (14, 16, 17). These approaches include *1*) increasing the ferritin-concentration cutoff to <30 μg/L for the entire sample or only in individuals with elevated inflammation (practitioners have interpreted the WHO recommendations on this approach both ways); *2*) excluding individuals with elevated inflammation; *3*) applying arithmetic correction factors (CFs); and *4*) using a regression correction (RC) approach. The first 2 approaches are currently recommended by the WHO, but there is a need to re-evaluate other approaches and reach a consensus on how to improve the estimates of the prevalence of depleted iron stores in populations with a high prevalence of inflammation (6).

The accurate assessment of iron deficiency is imperative to identify populations who are most in need of iron interventions (or not in need of additional iron) and to better understand the contributions of iron to other conditions including anemia. Therefore, in this article, the following 4 questions are explored: *1*) Is there a need to measure biomarkers of inflammation when measuring ferritin? *2*) Is there a need to measure >1 inflammation biomarker (CRP and AGP) to adjust ferritin? *3*) Is an additional adjustment needed to correct ferritin concentrations in the presence of malaria infection? and *4*) How do the different adjustment approaches for correcting for inflammation and for calculating the estimated prevalence of depleted iron stores compare with each other?

## METHODS

We used data from the Biomarkers Reflecting Inflammation and Nutritional Determinants of Anemia (BRINDA) project (www.BRINDA-nutrition.org) (19). The BRINDA protocol was reviewed by the institutional review boards of the NIH and was deemed to be non–human subjects research. The methods for identifying data sets, inclusion and exclusion criteria, and data management for the BRINDA project have been described in detail elsewhere (19). The surveys were nationally or regionally representative, and the inclusion criteria were surveys that were *1*) conducted after 2004, *2*) had target groups including preschool children (PSC), nonpregnant women of reproductive age (WRA), or both and *3*) measured ≥1 marker of iron (ferritin or soluble transferrin receptor) or vitamin A status (retinol binding protein or retinol) and ≥1 marker of inflammation (AGP or CRP). Surveys included in this analysis were those with measures of ferritin and inflammation (CRP or AGP). In all surveys in which WRA data were collected, PSC data were also collected as part of the same survey. Of the 16 PSC and 10 WRA BRINDA data sets, data from 15 PSC and 9 WRA surveys were applicable for analysis for this article. Malaria was measured in 5 PSC and 3 WRA data sets. Both CRP and AGP were measured in 8 of 15 PSC data sets (*n* = 13 surveys with CRP; *n* = 10 surveys with AGP) and 4 of 9 WRA data sets (*n* = 9 surveys with CRP; *n* = 4 surveys with AGP).

### Laboratory analysis

Venous or capillary blood was collected from each respondent, and plasma or serum was stored at −20°C until analysis; one survey used dried blood spots. Ferritin, AGP, and CRP concentrations were assessed with the use of a sandwich ELISA at the VitMin Laboratory in 8 data sets (Bangladesh, Cameroon, Cote d’Ivoire, Laos, Liberia, Kenya 2007 and 2010, and the Philippines) (20). Ferritin was measured with the use of an immunoassay in 7 data sets (Colombia, Georgia, Mexico 2006 and 2012, Nicaragua, Pakistan, and the United States). CRP was measured with the use of an immunoassay in one data set (United States), turbidimetry in 2 data sets (Colombia, Georgia), and nephelometry in 2 data sets (Mexico 2006 and 2012). AGP was measuring with the use of an immunoassay in Pakistan and turbidimetry in Nicaragua.

A comparison between the ELISA assay at the VitMin Laboratory and commercially available immunoassay assays has yielded high specificity and sensitivity and low intra-assay and interassay variability (20). The CDC laboratory also regularly conducts round-robin analyses to ensure comparability between the immunoassay and ELISA assay (D Whitehead, CDC, International Micronutrient Malnutrition Prevention and Control program, personal communication, 2016). In the few cases when turbidimetry and nephelometry were used, heterogeneity may have been of concern because of the lack of literature comparing these methods with the ELISA assay and immunoassay.

Current malaria infection was assessed with the use of microscopy in Kenya and Côte d’Ivoire (21), and current or recent malaria infection was assessed with the use of the Paracheck Pf rapid-diagnostic test (Orchid Biomedical System) in Liberia and histidine-rich protein 2 (Cellabs Pty Ltd.) in Cameroon. A standardizing malaria diagnostic was not used because a sensitivity analysis showed a minimal impact (22). The methods for identifying data sets, inclusion and exclusion criteria, and data management for the BRINDA project have been described in the methodologic overview in this supplement, which is an open access publication (23).

### Case definitions

Depleted iron stores were defined as concentrations of ferritin <12 μg/L in PSC and <15 μg/L in WRA. The WHO-defined concentration cutoff in the presence of inflammation of <30 μg/L was also applied in PSC and WRA (although not currently part of the WHO recommendations for WRA); the higher cutoff was applied to the entire sample as well as only in individuals with subclinical inflammation (6). Malaria infection was defined as either positive or negative. Inflammation was defined as a CRP concentration >5 mg/L, AGP concentration >1 g/L, or both (14). A household socioeconomic status (SES) asset score was defined by survey investigators who applied a principal component analysis within each survey to household characteristics and item ownership. The poorest wealth quintile was compared with the higher quintiles. In Georgia and the United States, where these data were unavailable, household employment was used in Georgia, and a poverty-index ratio of family income to family size for the comparison of lowest to higher levels was used in the United States. Maternal education was defined as any school compared with no school.

### Statistical analysis

Descriptive statistics were calculated with the use of STATA 12.0 software (StataCorp) and cross-checked with SAS 9.4 software (SAS Institute). Correlations between ferritin, CRP, and AGP concentrations and malaria infection were calculated with the use of Kendall’s τ coefficient with the use of the SOMERSD package (24). The Taylor linearization method was used to obtain unbiased estimates that incorporated the sampling weight, strata, and cluster (as applicable) when analyzing individual surveys. To combine data, individual survey analyses, accounting for the complex survey design, were performed with the use of the survey package in R 3.2.2 software (R Core Team) (25). Individual survey estimates were combined with the use of a meta-analysis approach with the use of the metafor package in R 3.2.2 software (26, 27). The heterogeneity of estimates across the surveys was assessed with the use of Cochran’s heterogeneity test (26, 27).

The prevalence of depleted iron stores (ferritin concentration <12 or <15 μg/L in WRA) was estimated without any adjustments to ferritin and was referred to as the unadjusted estimate. The following 4 approaches were used to adjust ferritin for inflammation and malaria: a higher ferritin-concentration cutoff of <30 μg/L, exclusion, CF, and RC (23).

### Higher ferritin cutoff approach

The higher ferritin-cutoff adjustment approach uses a higher ferritin-concentration cutoff of <30 μg/L. We tried 2 different approaches when the higher ferritin cutoff was used. We applied the cutoff to the entire sample as well as to the subset of individuals with elevated CRP and AGP (as defined by a CRP concentration >5 mg/L, AGP concentration >1 g/L, or both).

### Exclusion approach

The exclusion approach uses the inflammation, malaria-biomarker information, or both to exclude individuals with elevated inflammation (as defined by a CRP concentration >5 mg/L, AGP concentration >1 g/L, or both) or individuals with malaria infection from the analysis; this resulted in a smaller sample size. We excluded these individuals and calculated the estimated prevalence of depleted iron stores in the remaining individuals.

### CF approach

The CF approach, as proposed by Thurnham et al. (14), uses a arithmetic CF. We calculated the CF by grouping inflammation into the following 4 groups: *1*) reference (both CRP concentration ≤5 mg/L and AGP concentration ≤1 g/L); *2*) incubation (CRP concentration >5 mg/L and AGP concentration ≤1 g/L); *3*) early convalescence (both CRP concentration >5 mg/L and AGP concentration >1 g/L); and *4*) late convalescence (CRP concentration ≤5 mg/L and AGP concentration >1 g/L). We also calculated the CF by grouping inflammation or malaria into 2 groups in which CRP, AGP, or malaria were used independently. CFs were defined as the ratio of geometric means of the reference group (nonelevated CRP, AGP, or malaria negative depending on the grouping used) to those of the respective inflammation group or malaria group. CFs were calculated based on internal survey-specific data [termed the internal correction factor (ICF)], a previous meta-analysis with the use of distinct data from the BRINDA data set [termed the Thurnham correction factor (TCF)] (15), and the meta-analyzed BRINDA data set [termed the BRINDA correction factor (BCF)]. The CF approach was performed for CRP alone (termed ICF-CRP, TCF-CRP, BCF-CRP), AGP alone (termed ICF-AGP, TCF-AGP, BCF-AGP), and both CRP and AGP (termed ICF-CRP+AGP, TCF-CRP+AGP, BCF-CRP+AGP). CFs were further calculated for malaria with the use of the ICF approach only.

### RC approach

The RC approach uses linear regression to adjust ferritin concentrations by the CRP and AGP concentrations on a continuous scale and malaria infection as a dichotomous variable as defined in the BRINDA methods article (23). Briefly, the adjusted ferritin equation was calculated by subtracting the influence of CRP, AGP, and malaria as follows:


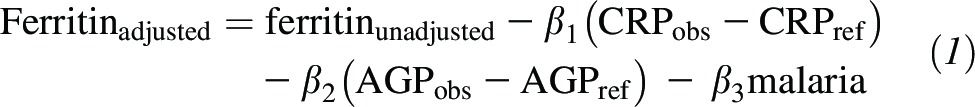


Depending on the available data, CRP, AGP, or malaria can be included in the model. β_1_ is the CRP regression coefficient, β_2_ is the AGP regression coefficient, β_3_ is the malaria regression coefficient, obs is the observed value, and ref is the external reference value generated to define low inflammation (the maximum values of the lowest CRP or AGP decile with the use of combined BRINDA data with nonlogged reference values as follows: CRP concentration in PSC: 0.10 mg/L; CRP concentration in WRA: 0.16 mg/L; AGP concentration in PSC: 0.59 g/L; AGP concentration in WRA: 0.54 g/L). CRP, AGP, and ferritin were all ln transformed; CRP and AGP were continuous variables, and malaria was a dichotomous variable. The correction was only applied to individuals with ln CRP greater than ln CRP_ref_ or ln AGP greater than ln AGP_ref_ to avoid overadjustments (23).

The first step in the RC approach was to ln transform ferritin, CRP, and AGP concentrations to approximate normality on the basis of regression diagnostics. CRP data contained values of zero, and thus, the survey-specific lowest value was used to replace zeros before applying the ln transformation. Second, linear regression coefficients for CRP, AGP, malaria, or a combination were estimated (bivariate and multivariate) with ferritin as the outcome. A test of multicollinearity between ln CRP and ln AGP or between malaria and ln CRP or ln AGP was assessed on the basis of a test of tolerance (>0.1) and a variance inflation factor (<5) to determine whether it was appropriate to include all variables in the model. Third, an ln-CRP reference value was subtracted from the ln-CRP value, and an ln-AGP reference value was subtracted from ln-AGP concentrations in the regression equation.

The RC approach is presented based on internal survey-specific data [termed the internal regression correction (IRC)] and the meta-analyzed BRINDA data [termed BRINDA regression correction (BRC)]. An illustrative example of how to adjust ferritin concentrations for inflammation with the use of the IRC approach on the basis of real data from Liberia in PSC is provided in **Supplemental Figure 1**. The BRC approach entailed the replacement of CRP and AGP β coefficients in the adjusted ferritin Equation *1* with the meta-analysis β coefficients. The same external reference values were used when applying both the IRC and BRC approaches. Each of these RCs was performed for CRP alone (termed IRC-CRP, BRC-CRP), AGP alone (termed IRC-AGP, BRC-AGP), or both CRP and AGP (termed IRC-CRP+AGP, BRC-CRP+AGP). The IRC was also performed for malaria alone, malaria plus CRP alone, malaria plus AGP alone, and malaria plus both CRP and AGP. The BRC approach was not applied when including malaria in the model because of the limited number of data sets that measured malaria.

We also tested whether potential confounders significantly influenced the relation between ferritin and inflammation. We added sex, age (months in PSC and years in WRA), maternal education, and SES to the model, and the CRP and AGP slopes that were adjusted for these confounders were extracted and used in the adjusted ferritin equation.

### Comparison of adjustments

Unadjusted and adjusted prevalence estimates of depleted iron stores were compared with the use of McNemar’s chi-square statistics; statistical significance was defined as *P* < 0.05 before applying the Bonferroni corrections to correct for multiple comparisons (*P* = 0.05 ÷ *k*, where *k* equals the number of comparisons). To examine whether malaria infection remained associated with estimated depleted iron stores after adjustment for CRP and AGP, we stratified the adjusted estimated prevalence of depleted iron stores (ferritin concentrations adjusted with the use of the IRC approach) by malaria status and compared the prevalence of individuals with and without malaria with the use of the adjusted Wald’s test to determine the effect of malaria in addition to elevated APPs. It was decided a priori that adjustments for CRP and AGP together and for CRP, AGP, and malaria combined would be explored irrespective of the significance between the malaria and nonmalaria estimated prevalences of depleted iron stores.

## RESULTS

### Participant characteristics

Our study sample was restricted to participants with no missing values for ferritin, CRP, AGP, or malaria (in surveys that measured malaria); this resulted in a total loss of 6.4% (1901 of the 29,766) of the observations that met the BRINDA inclusion criteria in PSC and of 3.4% (887 of 25,731) of the observations that met the BRINDA inclusion criteria in WRA. Participants who were excluded because of missing ferritin, CRP, AGP, or malaria data did not differ from those who were included with regard to sex, age, or SES (data not shown). The PSC mean age range was 8.3–41.5 mo, and there was considerable age-range variability notably for Bangladesh (6–11 mo), Liberia, Kenya 2007 and 2010 (6–35 mo), and the Philippines (6–23 mo), whereas the age range in the remaining 10 surveys was from either 6–59 mo (*n* = 5) or 12–59 mo (*n* = 5) (**Table 1**). In contrast, WRA age ranged from 15 to 49 y in all surveys with a mean age range of 27.2–34 y (Table 1). The prevalence of inflammation varied across surveys in PSC (CRP concentration >5 mg/L: 6.0–40.4%; AGP concentration >1 g/L: 21.2–64.5%) and WRA (CRP concentration >5 mg/L: 7.9–29.5%, AGP concentration >1 g/L: 7.2–26.9%) (Table 1). The prevalence of malaria varied by 13 percentage points (pps) in both PSC (19.7–32.5%) and WRA (5.0–17.9%) (Table 1).

**TABLE 1:** Age, inflammation, and malaria infection in preschool children and women of reproductive age: the BRINDA project^1^

Survey	*n*	Age^2^	CRP concentration >5 mg/L	AGP concentration >1 g/L	CRP concentration >5 mg/L or AGP concentration >1 g/L	Malaria
Preschool children						
Bangladesh	1493	8.3 (6–11)	14.3 (11.8, 16.7)	33.4 (29.9, 36.9)	35.8 (32.2, 39.5)	—
Cameroon	774	30.8 (12–59)	37.5 (32.7, 42.3)	39.3 (33.7, 45.0)	48.3 (43.1, 53.5)	25.9 (20.2, 31.5)
Colombia	3866	37.6 (12–59)	18.8 (17.1, 20.5)	—	—	—
Côte d’Ivoire	733	31.7 (6–59)	40.4 (36.5, 44.3)	64.5 (60.3, 68.6)	67.5 (63.8, 71.3)	27.2 (22.3, 32.0)
Georgia	2142	36.4 (12–59)	24.7 (21.8, 27.5)	—	—	—
Kenya 2007	888	19.9 (6–35)	27.8 (23.9, 31.7)	64.2 (60.2, 68.2)	66.0 (61.9, 70.1)	19.7 (15.8, 23.6)
Kenya 2010	843	21.4 (6–35)	34.2 (29.6, 38.7)	60.7 (56.0, 65.4)	61.9 (57.2, 66.6)	32.5 (28.4, 36.6)
Laos	481	33.1 (6–59)	16.6 (11.2, 22.1)	41.7 (33.9, 49.4)	44.0 (36.6, 51.5)	—
Liberia	1434	19.9 (6–35)	29.5 (26.5, 32.5)	56.2 (52.5, 60.0)	59.1 (55.6, 62.7)	29.4 (26.2, 32.6)
Mexico 2006	1590	41.5 (12–59)	11.2 (9.0, 13.4)	—	—	—
Mexico 2012	2538	37.5 (12–59)	11.7 (9.3, 14.1)	—	—	—
Nicaragua	957	33.3 (6–59)	—	26.9 (21.2, 32.5)	—	—
Pakistan	7221	27.3 (6–59)	—	35.5 (34.0, 37.0)	—	—
Philippines	1767	15.0 (6–23)	13.9 (11.6, 16.2)	21.2 (17.7, 24.6)	26.0 (22.4, 29.5)	—
United States 2003–2006	1138	37.9 (6–59)	6.0 (4.4, 7.5)	—	—	—
Women of reproductive age						
Cameroon	751	27.2 (15–49)	17.8 (14.8, 20.7)	7.2 (5.1, 9.3)	19.7 (16.6, 22.9)	15.0 (11.3, 18.6)
Colombia	9083	29.1 (15–49)	22.0 (20.8, 23.2)	—	—	—
Côte d’Ivoire	816	27.6 (15–49)	19.7 (16.5, 22.8)	26.9 (23.5, 30.4)	33.7 (29.6, 37.9)	5.0 (3.4, 6.5)
Georgia	1688	32.7 (15–49)	29.5 (26.6, 32.4)	—	—	—
Laos	816	29.3 (15–49)	7.9 (5.6, 10.2)	9.3 (7.1, 11.6)	13.9 (10.9, 16.8)	—
Liberia	1875	28.6 (15–49)	14.3 (12.1, 16.4)	10.4 (8.7, 12.2)	18.5 (16.2, 20.8)	17.9 (15.3, 20.4)
Mexico 2006	3020	31.3 (15–49)	24.3 (21.8, 26.8)	—	—	—
Mexico 2012	3612	34.0 (15–49)	20.7 (18.2, 23.3)	—	—	—
United States 2003–2006	3183	33.5 (15–49)	25.6 (23.5, 27.7)	—	—	—

1 Values are percentages (95% CIs) unless otherwise indicated. AGP, α-1-acid glycoprotein; BRINDA, Biomarkers Reflecting Inflammation and Nutritional Determinants of Anemia; CRP, C-reactive protein.

2 All values are means (minimum to maximums in parentheses). Age is given in whole months in children and in whole years in women.

### Relation between ferritin, inflammation, and malaria

The rank correlations between the 2 markers of the APR, CRP and AGP ranged from 0.48 to 0.63 in PSC and from 0.26 to 0.41 in WRA and were significant (*P* < 0.001) in all surveys (**Supplemental Table 1**). There was a moderately positive association between ferritin with CRP and AGP, and the strength of the relations were similar for both APPs and across PSC surveys (CRP range: −0.02 in Georgia to 0.44 in Kenya 2010; AGP range: 0.04 in Pakistan to 0.44 in Kenya 2010) except between ferritin and CRP in Georgia and between ferritin and AGP in Pakistan. The relation between ferritin with CRP and AGP was weaker in WRA than in PSC. The rank correlations between malaria (defined as positive or negative) and ferritin ranged from 0.14 to 0.25 in PSC and from >−0.01 to 0.07 in WRA (Supplemental Table 1). The rank correlations between malaria and CRP or AGP ranged from 0.12 to 0.27 in PSC and from 0.04 to 0.10 in WRA.

**FIGURE 1:**
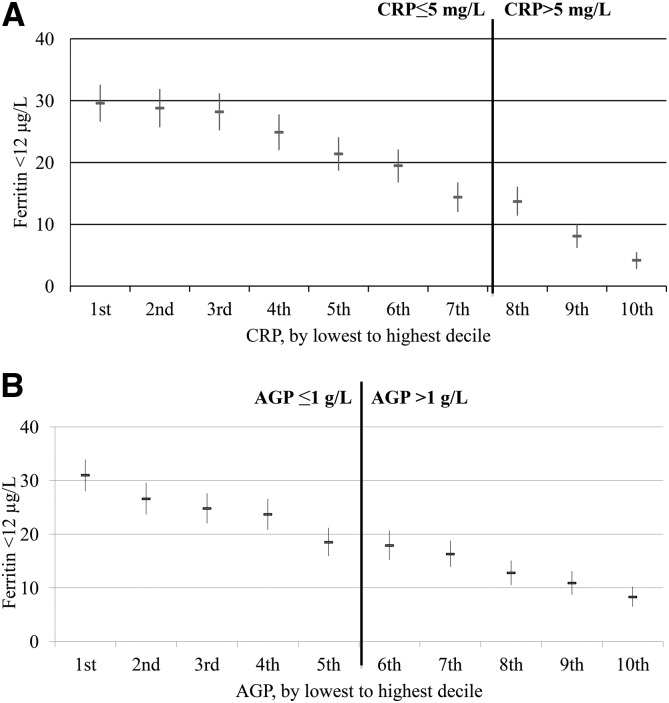
Estimated prevalence [% (95% CI)] of depleted iron stores in preschool children by CRP (A) and AGP (B) deciles: the Biomarkers Reflecting Inflammation and Nutritional Determinants of Anemia (BRINDA) project. The analysis was restricted to countries (Bangladesh, Cameroon, Côte d’Ivoire, Kenya 2007, Kenya 2010, Laos, Liberia, and Philippines) that measured both CRP and AGP for comparability between CRP and AGP relations with depleted iron stores. Sample size: *n* = 8458. Depleted iron stores were defined as a ferritin concentration <12 μg/L. Bold vertical lines indicate the commonly used cutoffs for CRP and AGP. AGP, α-1-acid glycoprotein; CRP, C-reactive protein.

**FIGURE 2:**
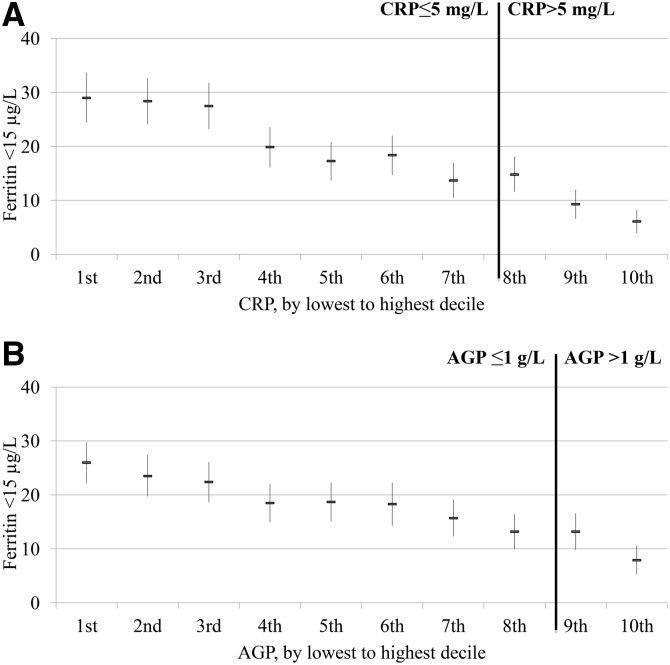
Estimated prevalence [% (95% CI)] of depleted iron stores in women of reproductive age by CRP (A) and AGP (B) deciles: the Biomarkers Reflecting Inflammation and Nutritional Determinants of Anemia (BRINDA) project. The analysis was restricted to countries (Cameroon, Côte d’Ivoire, Laos, and Liberia) that measured both CRP and AGP for comparability between CRP and AGP relations with depleted iron stores. Sample size: *n* = 4352. Depleted iron stores were defined as a ferritin concentration <15 μg/L. Bold vertical lines indicate the commonly used cutoffs for CRP and AGP. AGP, α-1-acid glycoprotein; CRP, C-reactive protein.

The relation between the estimated prevalence of depleted iron stores and inflammation deciles visually appeared to follow a linear pattern in PSC (**Figure 1**) and WRA (**Figure 2**) with the estimated prevalence in depleted iron stores incrementally increasing as the concentrations of CRP and AGP decreased. The relation between the estimated prevalence of depleted iron stores and inflammation declines did not vary according to whether subjects were above or below CRP and AGP cutoffs. In the lowest CRP decile group, the proportion of estimated depleted iron stores (ferritin concentration <12 μg/L) was substantially higher (29.6%) than in the highest CRP decile group (4.2%) in PSC; similarly, the estimated prevalence of depleted iron stores was markedly higher in the lowest AGP decile (31.0%) than in the highest AGP decile (8.3%). The prevalences between highest and lowest decile of inflammation for WRA followed the same pattern as for PSC; the estimated prevalence of depleted iron stores (ferritin concentration <15 μg/L) was 6.1% and 29.0% for the highest and lowest CRP decile groups, respectively, and 7.9% and 26.0% for the highest and lowest AGP decile groups, respectively.

### CFs for ferritin, inflammation, and malaria

The geometric mean of ferritin was lowest in individuals with no inflammation in PSC and WRA compared with in individuals with inflammation with and without malaria (as a dichotomous variable) (**Table 2**). Individuals who were stratified by malaria status (as a dichotomous variable) and by the 4-inflammation groups had the highest ferritin concentrations in the group with malaria and elevated CRP and AGP in PSC but not in WRA (Table 2). The CFs were <1 across all group combinations (CRP, AGP, and malaria) and surveys for both PSC and WRA. The TCFs that were derived from a previous meta-analysis in which all population groups were combined (incubation: 0.77; early convalescence: 0.53; late convalescence: 0.75), were similar to the BCFs although we showed the BCFs in PSC (incubation: 0.68; early convalescence: 0.38; late convalescence: 0.65) were lower than in WRA (incubation: 0.66; early convalescence: 0.52; late convalescence: 0.82).

**TABLE 2:** Ferritin in preschool children and women of reproductive age according to inflammation stage and malaria status: the BRINDA project^1^

	Malaria-endemic countries^2^			
	Malaria negative		Malaria positive	
Inflammatory stage	*n*	Ferritin, μg/L	*n*	Ferritin, μg/L
Preschool children				
Reference	1693	16.71 (11.6, 24.0)^3^	201	25.97 (18.9, 35.6)
Incubation	123	24.50 (16.0, 37.5)	37	38.59 (25.4, 58.8)
Early convalescence	669	40.28 (27.2, 59.6)	666	77.81 (66.8, 90.7)
Late convalescence	992	23.22 (16.9, 32.0)	291	41.05 (32.2, 52.3)
Women of reproductive age				
Reference	2401	31.07 (26.4, 36.6)	267	34.91 (25.7, 47.4)
Incubation	230	41.07 (34.3, 49.2)	76	47.26 (32.1, 69.5)
Early convalescence	190	59.12 (52.3, 66.8)	67	59.19 (48.5, 72.2)
Late convalescence	185	40.49 (31.4, 52.3)	26	39.84 (23.8, 66.7)

1 Reference is defined as a CRP concentration ≤5 mg/L and AGP concentration ≤1 g/L; incubation is defined as a CRP concentration >5 mg/L and AGP concentration ≤1 g/L; early convalescence is defined as a CRP concentration >5 mg/L and AGP concentration >1 g/L; and late convalescence is defined as an AGP concentration >1 g/L and CRP concentration ≤5 mg/L. AGP, α-1-acid glycoprotein; BRINDA, Biomarkers Reflecting Inflammation and Nutritional Determinants of Anemia; CRP, C-reactive protein.

2 Surveys had to have a marker for CRP, AGP, and malaria to be included and were as follows: Cameroon, Côte d’Ivoire, Kenya 2007, Kenya 2010, and Liberia (preschool children); and Cameroon, Côte d’Ivoire, and Liberia (women of reproductive age).

3 Geometric mean; 95% CI in parentheses (all such values).

### RC slopes for ferritin, inflammation, and malaria

Bivariate linear regression with ln ferritin as the outcome resulted in an unstandardized ln-CRP slope that ranged from <−0.01 (Georgia) to 0.31 (Côte d’Ivoire) and an ln-AGP slope from 0.20 (Philippines) to 1.84 (Kenya 2010) in PSC (**Supplemental Table 2**). In WRA, the unstandardized ln-CRP slope ranged from 0.06 (Colombia) to 0.19 (Mexico 2012) for CRP and from 0.45 (Laos) to 0.71 (Cameroon and Liberia) for AGP (**Supplemental Table 3**). There was no multicollinearity between ln CRP and ln AGP or between ln CRP, ln AGP, and malaria in the PSC and WRA models across surveys. A multivariate analysis of ln ferritin with both ln CRP and ln AGP in the model generally dampened the ln-CRP slope and ln-AGP slope in PSC and WRA (**Supplemental Table 4**) as did the inclusion of malaria (data not shown).

Heterogeneity was shown between the slopes that were meta-analyzed to obtain the external slope for the BRC approach. The test of residual heterogeneity QE (df = 21) = 645 and QE (df = 9) = 30 for PSC and WRA, respectively, when both CRP and AGP were in the model. The highest degree of heterogeneity was shown when creating a bivariate meta-analyzed AGP slope in PSC. Although all surveys were retained in the combined analyses, the removal of Pakistan from the model reduced the heterogeneity from QE (df = 18) = 1779 to QE (df = 16) = 593, although it was still significant (*P* < 0.001). Similarly, if Georgia was excluded from the WRA data when creating a bivariate meta-analyzed CRP slope, the heterogeneity changed from QE (df = 16) = 2358 to QE (df = 14) = 376. Neither Georgia nor Pakistan was included when creating the multivariate meta-analyzed CRP and AGP slopes because both surveys only included one marker of inflammation.

### Unadjusted prevalence of depleted iron stores

There was substantial variation in the prevalence of unadjusted depleted iron stores (ferritin concentration <12 μg/L in PSC and <15 μg/L in WRA) across surveys (**Table 3**). The prevalence range was wider in the surveys that measured either CRP or AGP (PSC range: 0.3–46.9%; WRA range: 1.4–27.9%) (Table 3) than in the surveys that measured both CRP and AGP (PSC range: 8.0–38.9%; WRA range: 12.9–22.7%) (Table 3). In the surveys in which data were available for both PSC and WRA, there was considerable variation in terms of which target group (PSC or WRA) had a higher prevalence of depleted iron stores (Table 3).

**TABLE 3:** Estimated prevalence of depleted iron stores unadjusted and after excluding subjects with inflammation in preschool children and women of reproductive age: the BRINDA project^1^

	Unadjusted		CRP concentration ≤5 mg/L^2^		AGP concentration ≤1 g/L^2^		CRP concentration ≤5 mg/L or AGP concentration ≤1 g/L^2^	
Survey	*n*	% (95% CI)	*n*	% (95% CI)	*n*	% (95% CI)	*n*	% (95% CI)
Preschool children								
Bangladesh	1493	8.0 (5.6, 10.3)	1280	8.9 (6.4, 11.4)	994	10.5 (7.7, 13.2)	958	10.6 (7.7, 13.5)
Cameroon	774	14.9 (12.1, 17.6)	493	20.2 (16.3, 24.1)	483	20.8 (17.0, 24.6)	411	21.6 (17.2, 26.0)
Colombia	3866	10.1 (8.8, 11.4)	3126	10.6 (9.1, 12.0)	—	—	—	—
Côte d’Ivoire	733	11.7 (8.9, 14.5)	429	17.1 (12.7, 21.4)	250	16.5 (11.4, 21.5)	227	18.0 (12.7, 23.4)
Georgia	2142	0.3 (<0.1, 0.5)	1646	0.1 (<0.1, 0.3)	—	—	—	—
Kenya 2007	888	38.9 (34.7, 43.0)	641	45.4 (40.8, 50.0)	318	53.1 (47.2, 59.1)	302	54.3 (48.4, 60.3)
Kenya 2010	843	19.2 (15.9, 22.6)	555	27.4 (23.1, 31.7)	331	34.1 (28.6, 39.6)	321	35.2 (29.6, 40.8)
Laos	481	16.6 (12.4, 20.7)	405	18.4 (14.1, 22.7)	287	19.2 (14.1, 24.4)	274	19.5 (14.3, 24.8)
Liberia	1434	20.4 (17.8, 23.0)	1059	26.1 (22.9, 29.3)	672	29.3 (24.9, 33.7)	633	30.6 (26.0, 35.1)
Mexico 2006	1590	23.4 (20.3, 26.6)	1412	24.9 (21.5, 28.4)	—	—	—	—
Mexico 2012	2538	13.5 (11.1, 15.9)	2276	14.8 (12.2, 17.5)	—	—	—	—
Nicaragua	957	33.2 (28.6, 37.9)	—	—	712	37.9 (32.8, 43.1)	—	—
Pakistan	7221	46.9 (45.4, 48.5)	—	—	4679	46.9 (45.0, 48.8)	—	—
Philippines	1767	26.2 (23.0, 29.4)	1500	28.8 (25.1, 32.4)	1345	30.0 (26.0, 33.9)	1274	30.8 (26.9, 34.7)
United States 2003–2006	1138	10.5 (7.9, 13.2)	1064	11.0 (8.3, 13.8)	—	—	—	—
Women of reproductive age								
Cameroon	751	12.9 (10.2, 15.6)	616	13.7 (10.7, 16.8)	698	13.1 (10.2, 15.9)	602	14.0 (10.9, 17.1)
Colombia	9083	22.9 (21.8, 24.1)	7064	24.1 (22.8, 25.4)	—	—	—	—
Côte d’Ivoire	816	13.6 (10.7, 16.4)	660	15.3 (11.7, 18.8)	599	16.2 (12.7, 19.7)	546	16.6 (12.8, 20.5)
Georgia	1688	1.4 (0.8, 2.1)	1216	1.6 (0.8, 2.4)	—	—	—	—
Laos	816	22.7 (17.5, 27.9)	752	24.0 (18.6, 29.5)	734	22.9 (17.3, 28.4)	698	23.8 (18.0, 29.5)
Liberia	1875	17.9 (15.5, 20.3)	1603	19.9 (17.1, 22.7)	1677	19.1 (16.5, 21.6)	1520	20.0 (17.2, 22.7)
Mexico 2006	3020	27.5 (24.6, 30.3)	2268	28.5 (25.4, 31.6)	—	—	—	—
Mexico 2012	3612	27.9 (24.9, 30.9)	2823	30.4 (27.0, 33.8)	—	—	—	—
United States 2003–2006	3183	13.1 (11.4, 14.8)	2408	14.7 (12.6, 16.8)	—	—	—	—

1 Depleted iron stores were defined as a ferritin concentration <12 μg/L for preschool children and <15 μg/L for women of reproductive age. AGP, α-1-acid glycoprotein; BRINDA, Biomarkers Reflecting Inflammation and Nutritional Determinants of Anemia; CRP, C-reactive protein.

2 Estimated prevalence of depleted iron stores in the subset of the sample with nonelevated inflammation.

### Adjusted estimated prevalence of depleted iron stores with the exclusion of subjects with inflammation to adjust ferritin for inflammation

The exclusion of PSC with elevated CRP or AGP resulted in a substantial sample-size loss of 26–68% in PSC and 14–34% in WRA (Table 3). In the surveys in which CRP was measured, the greatest absolute change in the estimated prevalence of depleted iron stores of subjects with CRP concentrations ≤5 mg/L compared with that of the full population was in Kenya 2010 (an estimated prevalence of 27.4% compared with 19.2%, respectively) for PSC and in Mexico 2012 (30.4% compared with 27.9%, respectively) for WRA (Table 3). In surveys in which AGP was measured, the greatest absolute change in the estimated prevalence of depleted iron stores of subjects with AGP concentrations ≤1 g/L compared with that of the full population was in Kenya 2010 (34.1% compared with 19.2%, respectively) in PSC and in Côte d’Ivoire (an estimated prevalence of 16.2% compared with 13.6%, respectively) in WRA (Table 3). The greatest absolute change in the estimated prevalence of depleted iron stores in subjects with CRP concentrations ≤5 mg/L and AGP concentrations ≤1 g/L (Table 3) compared with that in the full population was an estimated prevalence of 35.2% compared with 19.2%, respectively (Kenya 2010), in PSC and an estimated prevalence of 16.6% compared with 13.6%, respectively (Côte d’Ivoire), in WRA (Table 3).

### Adjusted estimated prevalence of depleted iron stores with the use of CRP or AGP to adjust ferritin for inflammation by the CF and RC adjustment approach

**FIGURE 3:**
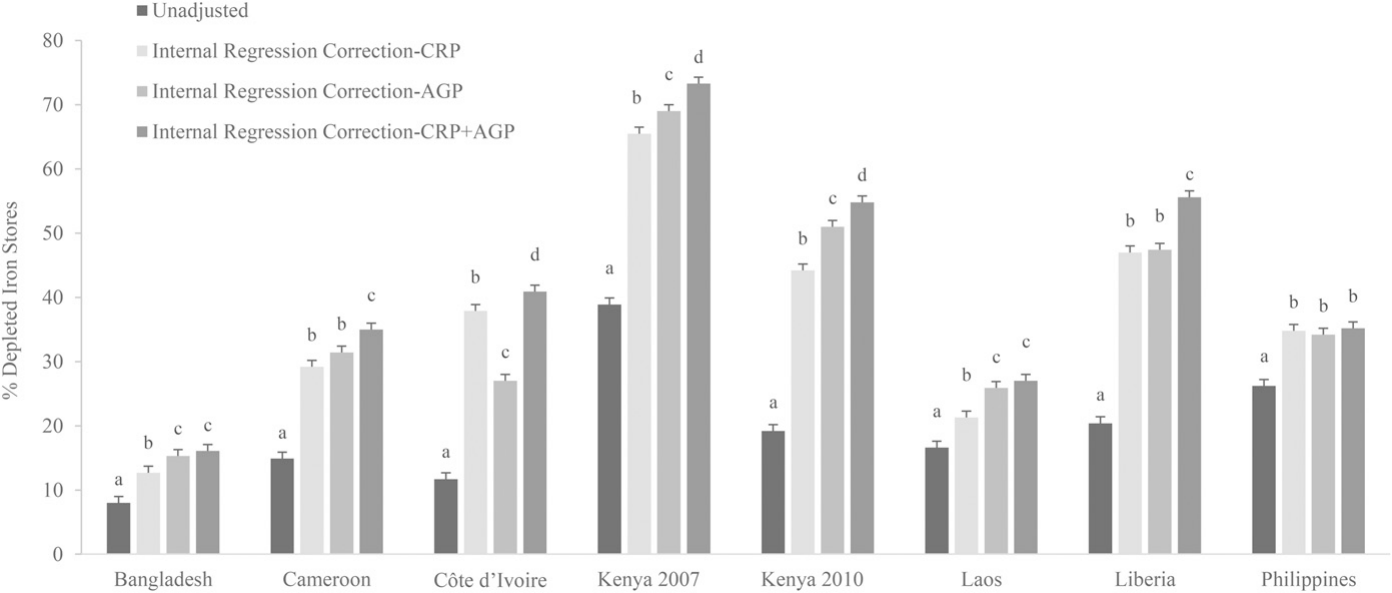
Estimated prevalence [% (95% CI)] of depleted iron stores on the basis of internal regression correction adjusting for CRP, AGP, or both in preschool children: Biomarkers Reflecting Inflammation and Nutritional Determinants of Anemia (BRINDA) project. Depleted iron stores were defined as a ferritin concentration <12 μg/L. Internal regression correction reference values were as follows: C-reactive protein = −2.26 ln(mg/L) [QE (df = 10) = 439.9, *P* < 0.0001]; and α-1-acid glycoprotein = −0.52 ln(g/L)] [QE (df = 10) = 584.6, *P* < 0.0001]. Bars without a common lowercase letter within a given survey differ, *P* < 0.05 (adjusted by using Bonferroni correction). AGP, α-1-acid glycoprotein; CRP, C-reactive protein.

The estimated prevalence of depleted iron stores in PSC incrementally increased compared with the unadjusted prevalence when CRP only, AGP only, and both CRP and AGP were used to adjust ferritin concentrations in general across all surveys and across adjustment methods (**Supplemental Tables 5–11**). For example, with the use of the ICF approach, the estimated prevalence of depleted iron stores in surveys that measured CRP and AGP resulted in an absolute median increase of 4.6 pps (range: 1.0–7.8 pps) when the ICF-CRP and of 5.9 pps (range: 2.9–17.0 pps) when the ICF-AGP approach was used compared with unadjusted values in PSC (Supplemental Table 7). The estimated prevalence of depleted iron stores with the use of the IRC approach compared with the unadjusted prevalence in surveys that measured CRP and AGP resulted in a higher absolute median increase when the IRC-CRP approach was used (19.7 pps; range: 4.7–26.6 pps) than when the IRC-AGP approach was used (15.9 pps; range: 7.3–31.8 pps); however, in most individual surveys, the absolute increase was greater when the IRC-AGP approach was used than when the IRC-CRP approach was used in PSC (**Figure 3**, Supplemental Table 10).

Similar to PSC, the estimated prevalence of depleted iron stores in WRA increased when correcting for CRP, AGP, or both (Supplemental Tables 5–11). However, the estimated prevalence of depleted iron stores in WRA in surveys that measured CRP and AGP was much lower than in PSC and showed an absolute median increase of 1.4 pps (range: 1.0–1.7 pps) with the use of the ICF-CRP approach and of 0.95 pps (range: 0.7–2.3 pps) with the use of the ICF-AGP approach compared with unadjusted values (Supplemental Table 7). The estimated prevalence of depleted iron stores in WRA in surveys that measured CRP and AGP showed an absolute median increase of 6.0 pps (range: 4.0–9.5 pps) with the use of the IRC-CRP approach and of 6.1 pps (range: 2.9–6.9 pps) with the use of the IRC-AGP approach compared with unadjusted values (Supplemental Table 10).

### Comparison of estimated prevalence of depleted iron stores with the use of different approaches to adjust ferritin for inflammation

The application of adjustments to ferritin concentrations, irrespective of the method, increased the estimated prevalence of depleted iron stores compared with unadjusted estimates; generally, there was a greater pp difference in PSC than in WRA (Supplemental Tables 5–11). The use of a higher ferritin-concentration cutoff of <30 mg/L for all PSC (regardless of inflammation status) resulted in large absolute increase in estimates of depleted iron stores upwards of 35 pps compared with unadjusted values (Supplemental Table 5). All other adjustments that required the use of inflammation data and comparisons between the different approaches described in this section are limited to adjustments for both CRP and AGP. Adjustments with the use of only CRP or AGP are shown in Supplemental Tables 6–11.

**FIGURE 4:**
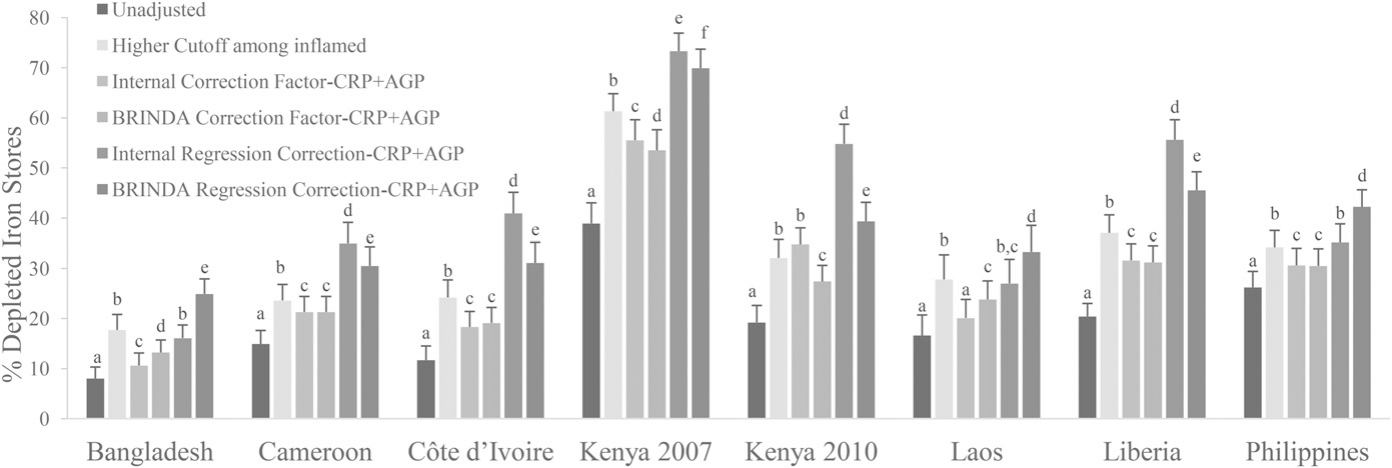
Estimated prevalence [% (95% CI)] of depleted iron stores with the use of different inflammation-adjustment approaches in preschool children: BRINDA project. Depleted iron stores were defined as a ferritin concentration <12 μg/L except in the higher-cutoff approach in which depleted iron stores were defined as a ferritin concentration <30 μg/L. Only surveys that measured both AGP and CRP are presented. Bars without a common lowercase letter within a given survey differ, *P* < 0.05 (adjusted by using Bonferroni correction). BRINDA correction factors were as follows—incubation phase: 0.68 (95% CI: 0.60, 0.79); early convalescence phase: 0.38 (95% CI: 0.30, 0.49); and late convalescence phase: 0.65 (95% CI: 0.58, 0.74) [QE (df = 28) = 515.9, *P* < 0.0001]. Internal regression correction and BRINDA regression correction reference values were as follows—CRP: −2.26 ln(mg/L) [QE (df = 10) = 439.9, *P* < 0.0001]; and AGP: −0.52 ln(g/L) [QE (df = 10) = 584.6, *P* < 0.0001]. BRINDA regression correction coefficients were as follows—ln CRP = 0.12 and ln AGP = 0.71 [QE (df = 21) = 644.8, *P* < 0.0001]. AGP, α-1-acid glycoprotein; BRINDA, Biomarkers Reflecting Inflammation and Nutritional Determinants of Anemia; CRP, C-reactive protein; -CRP+AGP, adjusting for C-reactive protein and α-1-acid glycoprotein.

**FIGURE 5:**
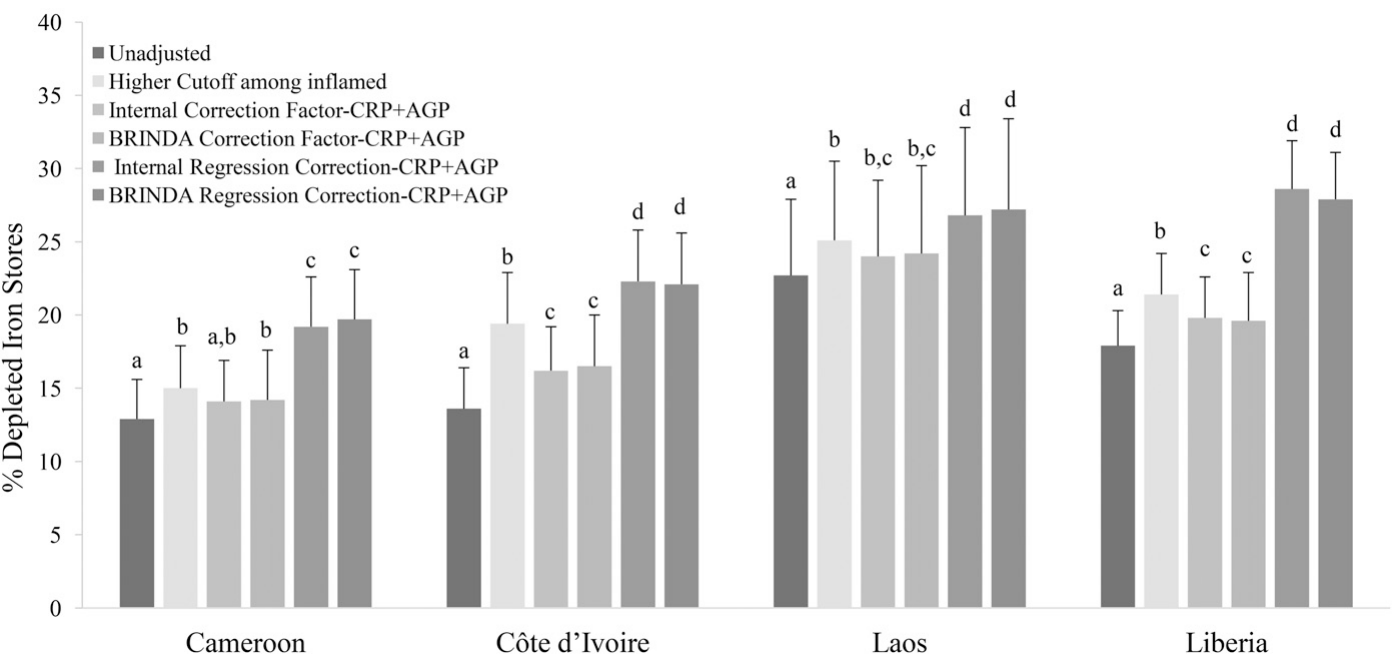
Estimated prevalence [% (95% CI)] of depleted iron stores with the use of different inflammation-adjustment approaches in women of reproductive age: BRINDA project. Depleted iron stores were defined as a ferritin concentration <15 μg/L except in the higher-cutoff approach in which depleted iron stores were defined as a ferritin concentration <30 μg/L. Only surveys that measured both AGP and CRP are presented. Bars without a common lowercase letter within a given survey differ, *P* < 0.05 (adjusted by using Bonferroni correction). BRINDA correction factors were as follows—incubation phase: 0.66 (95% CI: 0.53, 0.83); early convalescence phase: 0.52 (95% CI: 0.44, 0.61); and late convalescence phase: 0.82 (95% CI: 0.68, 0.99) [QE (df = 12) = 38.7, *P* < 0.0001]. Internal regression correction and BRINDA regression correction reference values were as follows—CRP: −1.83 ln(mg/L) [QE (df = 6) = 1142.4, *P* < 0.001]; and AGP-0.63 ln(g/L) [QE (df = 4) = 83.8, *P* < 0.0001]. BRINDA regression correction coefficients were as follows—ln CRP = 0.11; and ln-AGP: 0.34 [QE (df = 9) = 29.6, *P* < 0.001]. AGP, α-1-acid glycoprotein; BRINDA, Biomarkers Reflecting Inflammation and Nutritional Determinants of Anemia; CRP, C-reactive protein; -CRP+AGP, adjusting for C-reactive protein and α-1-acid glycoprotein.

With the use of a higher cutoff (ferritin concentration <30 mg/L) in PSC with elevated inflammation (CRP concentration >5 mg/L or AGP concentration >1 g/L) resulted in an absolute median increase of 12.2 pps (range: 8.0–22.4 pps) in the estimated prevalence of depleted iron stores compared with unadjusted values (**Figure 4**, Supplemental Table 6) and a much lower increase in WRA (absolute median: 3.0 pps; range: 2.1–5.8 pps) (**Figure 5**, Supplemental Table 6). Prevalences that were generated with the use of the higher cutoff in individuals with elevated inflammation minus the prevalences that were generated with the use of the ICF-CRP+AGP approach resulted in a lower estimated prevalence of depleted iron stores (absolute median difference: −5.6 pps; range: −7.1 to 2.7 pps) and, with the use of the IRC-CRP+AGP approach, resulted in a higher estimated prevalence of depleted iron stores (absolute median difference: 11.7 pps; range: −1.6 to 22.7 pps) in PSC. When nonmalaria and malaria countries were examined separately, the IRC-CRP+AGP was similar (absolute median difference: −0.8 pps; range: −1.6 to 1 pps) to the higher cutoff that was applied to individuals with elevated inflammation in nonmalaria countries, but not in the malaria countries (absolute median difference: 16.7 pps; range: 11.4–22.7 pps) in PSC. The difference between the higher cutoff in individuals with elevated inflammation resulted in a lower difference (absolute median difference: = −1.4 pps; range: −0.9 to −3.2 pps) and a higher difference (absolute median difference: 3.7 pps; range: 2.1–6.5 pps) than was shown with the use of the ICF-CRP+AGP and IRC-CRP+AGP approaches, respectively, in WRA.

When the ICF-CRP+AGP, BCF-CRP+AGP, and TCF-CRP+AGP adjustment approaches were applied to ferritin concentrations, the estimated prevalence of depleted iron stores increased by an absolute median difference of 6.5 pps (range: 2.6–16.6 pps), 7.3 pps (range: 4.3–14.6 pps), and 4.0 pps (range: 1.7–9.7 pps), respectively, compared with unadjusted values in PSC (Figure 4, Supplemental Tables 7–9). In contrast, the increase in the estimated prevalence in WRA was much lower [ICF-CRP+AGP absolute median difference: 1.6 pps (range: 1.2–2.6 pps); BCF-CRP+AGP absolute median difference: 1.6 pps (range: 1.3–2.9 pps); and TCF-CRP+AGP absolute median difference: 1.5 pps (range: 0.8–2.7 pps)] compared with unadjusted values (Figure 5, Supplemental Tables 7–9). The difference between the TCF-CRP+AGP and the BCF-CRP+AGP adjusted estimates of depleted iron stores was consistently lower by a small amount (absolute median difference: −3 pps; range: −2.6 to 4.9 pps), although in 2 surveys (Laos and Liberia), the difference between TCF and BCF was close to 5 pps in PSC (Supplemental Tables 8 and 9). The difference between the TCF-CRP+AGP and BCF-CRP+AGP approaches was negligible in WRA with a difference of <1 pp (Supplemental Tables 8 and 9).

The use of the IRC-CRP+AGP approach to adjust ferritin concentrations in PSC increased the estimated prevalence of depleted iron stores by an absolute median of 24.7 pps (range: 8.1–35.6 pps) compared with unadjusted values, and the use of the BRC-CRP+AGP approach to adjust ferritin concentrations in PSC increased the estimated prevalence of depleted iron stores by an absolute median of 18.0 pps (range: 16.0–31.0 pps) compared with unadjusted values, whereas in WRA, the prevalences were lower with absolute medians of 7.5 pps (range: 4.5–10.7 pps) and 7.7 pps (range: 4.5–10 pps), respectively compared with unadjusted values (Figures 4 and , Supplemental Tables 10 and 11). The comparison of differences between the use of the IRC-CRP+AGP approach compared with the BRC-CRP+AGP approach to adjust ferritin concentrations resulted in an absolute median difference of −3.4 pps (range: −16.5 to 8.8 pps) in PSC (Figure 4). The pp difference between IRC-CRP+AGP compared with BRC-CRP+AGP approaches was <1 pp in WRA (Figure 5, Supplemental Tables 10 and 11). The difference between the IRC-CRP+AGP– minus the ICF-CRP+AGP–adjusted depleted iron-store estimates was consistently higher [absolute median differences: 15.8 pps (range: 4.6–24 pps) and 5.8 pps (range: 3.2–8.1 pps)] in PSC and WRA, respectively (Figures 4 and ).

### Comparison of estimated prevalence of depleted iron stores with the use of different approaches to adjust ferritin for inflammation and malaria

**FIGURE 6:**
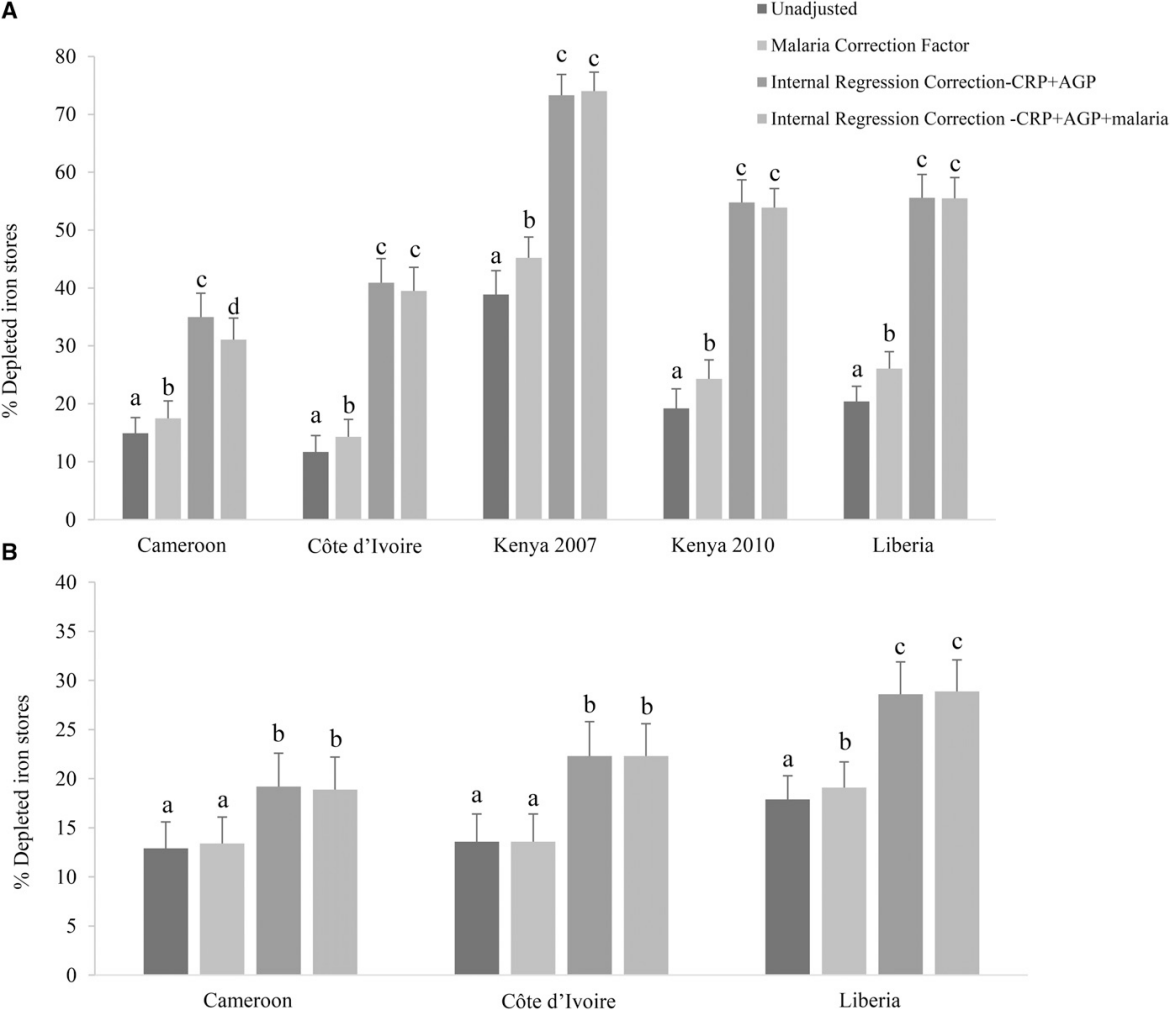
Estimated prevalence [% (95% CI)] of depleted iron stores with the use of different inflammation- and malaria-adjustment approaches in preschool children (A) and women of reproductive age (B): Biomarkers Reflecting Inflammation and Nutritional Determinants of Anemia (BRINDA) project. Depleted iron stores were defined as a ferritin concentration <12 μg/L in preschool children and a ferritin concentration <15 μg/L in women of reproductive age. Only surveys that measured malaria are presented. Bars without a common lowercase letter within a given survey differ, *P* < 0.05 (adjusted by using Bonferroni correction). -CRP+AGP, adjusting for C-reactive protein and α-1-acid glycoprotein; -CRP+AGP+malaria, adjusting for C-reactive protein, α-1-acid glycoprotein, and malaria.

Adjustment for malaria alone with the use of an ICF approach increased the estimated prevalence of depleted iron stores by an absolute median of 5.1 pps (range: 2.6–6.3 pps) in PSC and of 0.5 pps (range: 0–1.2 pps) in WRA (**Supplemental Table 12**). After the adjustment of ferritin concentrations with the use of the IRC-CRP+AGP approach, the estimated prevalence of depleted iron stores was significantly different (*P* < 0.0001) in subjects with and without malaria in 4 of 5 surveys that included malaria in PSC and in 1 of 3 surveys that included malaria in WRA (**Figure 6**, Supplemental Table 12). Adjustment of CRP and AGP alone (IRC-CRP+AGP) resulted in similar point estimates of depleted iron stores to those when adjusting for CRP and AGP plus malaria with the use of the IRC approach [absolute median differences: −0.9 pp (range: −3.9 to 0.7 pps) in PSC and 0 pps (range: −0.3 to 0.3 pps) in WRA)] (Figure 6, Supplemental Table 12).

### Estimated prevalence of depleted iron stores with the use of the IRC approach to adjust ferritin for inflammation taking into account confounders

There was little difference between the prevalence of depleted iron stores adjusting for ferritin with the use of the IRC approach when controlling for SES, maternal education, age, and sex (maternal education data were unavailable in Bangladesh, Liberia, Georgia, Mexico, and the United States for PSC and in all surveys for WRA, and SES data were unavailable in Bangladesh and Nicaragua). The absolute median difference of adjusted depleted iron stores controlling for confounders compared with not controlling for confounders was 0.40 pps (range: −1.2 to 3.8 pps) for IRC-CRP, 0.45 pps (range: −1.4 to 2.8 pps) for IRC-AGP, and 0.05 pps (range: −1.9 to 2.5 pps) for IRC-CRP+AGP in PSC (data not shown). The absolute median difference of depleted iron stores compared with not controlling for these factors was −0.90 pps (range: −3.7 to 0.2 pps) for IRC-CRP, 0.05 pps (range: −0.2 to 0.3 pps) for IRC-AGP, and −0.35 pps (range: −1.6 to 0 pps) for IRC-CRP+AGP in WRA (data not shown).

## DISCUSSION

The results of this multicountry analysis showed that inflammation and malaria infection affected ferritin concentrations such that individuals with inflammation or malaria infection had a higher ferritin concentration and were less likely to be classified as having depleted iron stores than were subjects without inflammation or malaria.

In this study, the first question we sought to answer was whether it is necessary to measure biomarkers of inflammation to estimate the prevalence of depleted iron stores. We showed a consistent positive correlation between ferritin with CRP and AGP concentrations, and the strength of the association was similar for both biomarkers; ferritin concentrations were highest when CRP and AGP were elevated (23) as have been shown by others (14). This finding lends support to the hypothesis that the magnitude of change in ferritin concentrations differs depending on the stage in the inflammatory process (18). Perhaps, more importantly, we showed that ferritin concentrations were altered well below the CRP and AGP cutoffs. This result indicates that clinically defined cutoffs for CRP and AGP may have less relevance when attempting to capture the influences of CRP and AGP on ferritin concentrations (23). These results corroborate findings by Duncan et al. (28), whereby low concentrations of CRP effected the concentrations of micronutrient biomarkers.

Our second question was whether both CRP and AGP are required to interpret ferritin concentrations. CRP tends to rise quickly in response to injury or infection and has a half-life of ∼2 d, whereas AGP rises slower but remains elevated with a half-life of ≥5 d (18). Not surprisingly, as has been shown elsewhere (29), adjustments with the use of both CRP and AGP increased the estimated prevalence of depleted iron stores compared with the use of either CRP or AGP alone, although AGP frequently provided similar estimates to those when adjusting for CRP and AGP. Nevertheless, CRP is more routinely measured and should continue to be measured along with AGP until we better understand the relation between ferritin, CRP, and AGP.

In answer to our third question, malaria infection was independently associated with ferritin after controlling for CRP and AGP. This result could reflect impaired iron incorporation in response to malaria infection by the suppression of erythropoietin (11–13). Alternatively, or in addition, it may be the effect of residual confounding of malaria-associated inflammation that are not fully captured by CRP or AGP (30). However, malaria adjustments alone resulted in lower adjustments to CRP and AGP, which were consistent with past studies (17). When CRP and AGP are measured, there appears to be limited utility in measuring malaria status to adjust ferritin concentrations.

Our fourth question was to compare adjustment approaches to estimate the prevalence of depleted iron stores in populations with inflammation. To compare approaches, we considered the following 3 criteria: *1*) precision, *2*) variability reflecting relation between nutrient biomarkers and severity of inflammation, and *3*) feasibility to implement across countries. The application of different adjustment approaches resulted in a high degree of variability in the estimated prevalence of depleted iron stores. The exclusion approach resulted in a loss of precision (averaging a 50% sample loss when excluding subjects with elevated CRP and AGP) and may have introduced bias. The use of a fixed ferritin-concentration cutoff of <30 μg/L (in subjects with elevated CRP or AGP) was relatively equivalent to the IRC approach in nonmalaria settings, whereas in malaria settings, the IRC approach resulted in a greater estimated prevalence of depleted iron stores. The fixed higher cutoff is a crude but easy adjustment approach, and its sensitivity and specificity should be further assessed because it was derived from a single study in pregnant women (1, 31, 32).

The ICF approach compared with the TCF or BCF approach has the advantage of using the inflammation profile that is specific to the data being adjusted. However, the precision of the ICF approach is lower because the estimated prevalence of depleted iron stores is based on the sample size of the reference group. Nevertheless, only in circumstances when the sample size is not sufficient to calculate a reliable ICF, an external CF should be considered. In our analysis, the BCFs were lower in children than in women. We suggest the use of the age-specific BCF instead of the TCF that was derived from a previous meta-analysis, which combined pregnant and nonpregnant women, men, children, and HIV-positive adults (14).

We showed that the IRC approach had promise because it better reflected the relation of ferritin concentrations with inflammation than the categorical approaches. Unlike in our study, in which we showed larger changes in the estimated prevalence of depleted iron stores after making IRC adjustments, other authors showed limited differences between a regression and categorical approach (16). These disparate results were likely due to previous studies having applied adjustments only to individuals above the cutoffs for CRP and AGP (16). We also showed that there was a high degree of variability in the strength of the linear relation (e.g., slopes) between ferritin and CRP and AGP across surveys, and thus, internal slopes (i.e., ICF approach) should be used instead of an external slope (i.e., BRC approach). The variation in slopes across surveys necessitates further investigations to identify potential modifiers of the ferritin–CRP-AGP relation in particular the examination of differential effects by the type of infection or inflammatory event.

We showed a stronger relation between inflammatory and ferritin biomarkers in children than in women, which translated into a greater increase in the estimated prevalence of depleted iron stores in children when adjusted for inflammation. This result may be indicative of differences in the response of ferritin to an inflammatory event across the life cycle. Although this hypothesis needs to be further explored, there has been supporting evidence of age-specific differences when it comes to iron metabolism (33), malaria (34), and pharmacokinetics (35).

The principal limitations of this study are the selection of data sets on the basis of convenience (i.e., the availability of data from BRINDA partners), the cross-sectional nature of the data, and the absence of a gold-standard measure of iron status. With the use of the BRINDA database, we were unable to determine whether CRP and AGP incompletely capture the relation between ferritin and inflammation or, alternatively, whether the adjustment approaches overadjust ferritin concentrations on the basis of a third unknown confounder. A comparison of adjustment approaches against a gold standard (e.g., bone marrow iron) and the use of longitudinal data would further contribute to the evidence.

An exploration of the potential utility of other iron biomarkers that are less affected by inflammation and infections would be worthwhile. One such biomarker, soluble transferrin receptor, was also shown to be influenced by inflammation and infections both individually and as part of the body iron equation with the use of a similar adjustment approach (36, 37). In addition, ferritin may also be elevated in the presence of genetic disorders (38) and obesity (39), and adjustments for these factors should be further studied.

In conclusion, the accurate assessment of iron status is critical to guide policy and program decisions. This study is meant as a step forward in identifying approaches to adjust ferritin concentrations in populations with high levels of inflammation. Our findings lend support for the use of the IRC approach. The use of this type of approach is not new to the field and has been widely adopted to adjust hemoglobin concentrations for altitude (40). However, the use of the IRC approach often results in a substantial increase in the estimated prevalence of depleted iron stores, which, in some cases, has been by a magnitude of 2- to 3-fold. Therefore, it will be important to describe the IRC approach to decision-makers or other audiences without an epidemiologic background to facilitate its use. The development of an automatic macro would also increase the usability of the IRC approach. This study contributes to the evidence on how to adjust ferritin concentrations for inflammation.
